# An integrated method for taxonomic identification of microorganisms

**DOI:** 10.18699/VJ20.630

**Published:** 2020-07

**Authors:** Yu.E. Uvarova, A.V. Bryanskaya, A.S. Rozanov, V.N. Shlyakhtun, E.A. Demidov, K.V. Starostin, T.N. Goryachkovskaya, S.V. Shekhovtsov, N.M. Slynko, S.E. Peltek

**Affiliations:** Institute of Cytology and Genetics of Siberian Branch of the Russian Academy of Sciences, Novosibirsk, Russia Kurchatov Genomic Center of the Institute of Cytology and Genetics of Siberian Branch of the Russian Academy of Sciences, Novosibirsk, Russia; Institute of Cytology and Genetics of Siberian Branch of the Russian Academy of Sciences, Novosibirsk, Russia Kurchatov Genomic Center of the Institute of Cytology and Genetics of Siberian Branch of the Russian Academy of Sciences, Novosibirsk, Russia; Institute of Cytology and Genetics of Siberian Branch of the Russian Academy of Sciences, Novosibirsk, Russia Kurchatov Genomic Center of the Institute of Cytology and Genetics of Siberian Branch of the Russian Academy of Sciences, Novosibirsk, Russia; Institute of Cytology and Genetics of Siberian Branch of the Russian Academy of Sciences, Novosibirsk, Russia Kurchatov Genomic Center of the Institute of Cytology and Genetics of Siberian Branch of the Russian Academy of Sciences, Novosibirsk, Russia; Institute of Cytology and Genetics of Siberian Branch of the Russian Academy of Sciences, Novosibirsk, Russia Kurchatov Genomic Center of the Institute of Cytology and Genetics of Siberian Branch of the Russian Academy of Sciences, Novosibirsk, Russia; Institute of Cytology and Genetics of Siberian Branch of the Russian Academy of Sciences, Novosibirsk, Russia Kurchatov Genomic Center of the Institute of Cytology and Genetics of Siberian Branch of the Russian Academy of Sciences, Novosibirsk, Russia; Institute of Cytology and Genetics of Siberian Branch of the Russian Academy of Sciences, Novosibirsk, Russia Kurchatov Genomic Center of the Institute of Cytology and Genetics of Siberian Branch of the Russian Academy of Sciences, Novosibirsk, Russia; Institute of Cytology and Genetics of Siberian Branch of the Russian Academy of Sciences, Novosibirsk, Russia Kurchatov Genomic Center of the Institute of Cytology and Genetics of Siberian Branch of the Russian Academy of Sciences, Novosibirsk, Russia; Institute of Cytology and Genetics of Siberian Branch of the Russian Academy of Sciences, Novosibirsk, Russia Kurchatov Genomic Center of the Institute of Cytology and Genetics of Siberian Branch of the Russian Academy of Sciences, Novosibirsk, Russia; Institute of Cytology and Genetics of Siberian Branch of the Russian Academy of Sciences, Novosibirsk, Russia Kurchatov Genomic Center of the Institute of Cytology and Genetics of Siberian Branch of the Russian Academy of Sciences, Novosibirsk, Russia

**Keywords:** identification of microorganisms, biochemical characteristics of bacteria, chemosystematics, mass spectrometric analysis, идентификация микроорганизмов, биохимические характеристики бактерий, хемосистематика, масс-спектрометрический анализ

## Abstract

For accurate species-level identification of microorganisms, researchers today increasingly use a combination of standard microbiological cultivation and visual observation methods with molecular biological and genetic techniques that help distinguish between species and strains of microorganisms at the level of DNA or RNA molecules. The aim of this work was to identify microorganisms from the ICG SB RAS Collection using an integrated approach that involves a combination of various phenotypic and genotypic characteristics. Key molecular-genetic and phenotypic characteristics were determined for 93 microbial strains from the ICG SB RAS Collection. The strains were characterized by means of morphological, physiological, moleculargenetic, and mass-spectrometric parameters. Specific features of the growth of the strains on different media were determined, and cell morphology was evaluated. The strains were tested for the ability to utilize various substrates. The strains studied were found to significantly differ in their biochemical characteristics. Physiological characteristics of the strains from the collection were identified too, e.g., the relationship with oxygen, type of nutrition, suitable temperature and pH ranges, and NaCl tolerance. In this work, the microorganisms analyzed were combined into separate groups based on the similarities of their phenotypic characteristics. This categorization, after further refinement and expansion of the spectrum of taxa and their metabolic maps, may serve as the basis for the creation of an “artificial” classification that can be used as a key for simplified and quicker identification and recognition of microorganisms within both the ICG SB RAS Collection and other collections

## Abstract (ru)

Для точной видовой идентификации микроорганизмов сегодня все чаще применяют сочетание стандартных микробиологических методов культивирования и визуального наблюдения с методами молекулярной биологии и генетики, помогающими различать виды и штаммы микроорганизмов на уровне молекул ДНК или РНК. Целью данной работы было проведение идентификации микроорганизмов из Коллекции ИЦиГ СО РАН с помощью комплексного подхода, сочетающего использование широкого спектра фенотипических и генотипических признаков. Для 93 штаммов микроорганизмов Коллекции ИЦиГ СО РАН описаны ключевые молекулярно-генетические и фенотипические свойства. Рассмотрены морфологические, физиологические, молекулярно-генетические и масс-спектрометрические характеристики штаммов. Установлены особенности роста штаммов на разных средах, изучена морфология клеток. Штаммы протестированы на способность использовать различные субстраты. Обнаружено, что исследованные штаммы значительно различались по своим биохимическим признакам. Определены физиологические особенности штаммов коллекции: отношение к кислороду, тип питания, диапазон температур и рН, отношение к NaCl и др. Исследованные микроорганизмы объединены в отдельные группы на основании сходства их фенотипических характеристик, что может при дальнейшей доработке и расширении спектра таксонов и их метаболических карт послужить основой для создания «искусственной» классификации, которая может быть использована в качестве ключа для упрощенной и более быстрой идентификации и распознавания микроорганизмов в рамках как Коллекции ИЦиГ СО РАН, так и других коллекций.

## Introduction

Identification of prokaryotes, which are morphologically less diverse than eukaryotes, is based on a wide variety of phenotypic – and in many cases also genotypic – characteristics. During the description and identification of bacteria, researchers study their cultivation properties, morphology, cell organization, physiological and biochemical features, chemical composition of cells, GC content of DNA, nucleotide sequence of the gene coding for 16S ribosomal RNA (rRNA), and other phenotypic and genotypic characteristics. 

Phenotypic methods of identification are popular mostly because of their relatively low cost. Phenotypic reactions usually include the responses to various chemical compounds or biochemical markers. Nonetheless, the manifestation of phenotypic traits of a microorganism – e. g., cell size and shape, sporulation, cell composition, antigenicity, biochemical activity, and sensitivity to antimicrobial agents – often depends on the nutrient media and culture conditions being used. Therefore, in recent years, to improve the classic methods of biochemical identification, investigators developed modern methods of biochemical identification (Church, 2016; Reyes, 2018). 

Characteristic features of the growth of microorganisms on solid and liquid nutrient media are categorized as cultivationrelated or macromorphological properties. Morphological characteristics and bacterial-cell organization include such traits as cell shape and size, cell motility, the presence of flagella, flagellation type, and sporogenesis capacity. In bacterial systematics, the top priority is given to Gram staining and cell wall structure.

Research on physiological and biochemical properties primarily includes determination of the nutrition mode of the bacterium being analyzed (photo-/chemo- and auto-/heterotrophy) and the type of energy metabolism (capacity for fermentation, aerobic or anaerobic respiration, or photosynthesis). It is important to identify such traits as the relationship of this bacterium with molecular oxygen, temperature, pH of the medium, with salinity, illuminance, and other environmental factors. This group of traits also contains the list of substrates utilized as sources of carbon, nitrogen, and sulfur; requirements for vitamins and other growth factors; formation of characteristic products of metabolism; and the expression of certain enzymes. For this purpose, special assays are often performed.

Many assays employed for the detection of the aforementioned traits (they are sometimes called “routine assays”) are crucial for clinical diagnoses and are widely used in medical microbiology. These assays require substantial time, a large number of complicated media and reagents, compliance with standardized operating procedures, and meticulous execution. To accelerate and facilitate the identification of some microorganisms, mostly those that are clinically important, researchers have developed various assay kits, such as MIKROLATEST® ID | Erba Lachema s.r.o. and BioLog. For instance, the MIKROLATEST® ID assay is designed for the identification of enterobacteria and represents a plastic chamber with wells containing colored diagnostic media. Whether the result is positive or negative is determined by changes in the color of a medium or by a reaction after the addition of special reagents (e.g., the assay of indole production and the Voges–Proskauer test).

A state-of-the-art phenotypic technology called BioLog yields valuable information about the properties of strains in addition to species level identification. Molecular techniques such as 16S rRNA sequencing and matrix-assisted laser desorption/ionization time of flight (MALDI-TOF) mass spectrometry do not provide information about the properties of a strain. The technology of carbon source utilization in the BioLog assay allows environmental microorganisms and pathogenic microorganisms to be identified by compiling a characteristic profile or “metabolic fingerprint” after certain assay reactions carried out in a microtiter plate. Suspension cultures are tested by means of a panel of preselected assays, then are incubated and analyzed on a signal reader, and the results are queried against databases.

Among the modern methods of biochemical identification is MALDI-TOF mass spectrometry, which is one of the latest methodologies for microbial identification. Even though this assay is “phenotypic,” it in a sense eliminates the gap in the reliability of the test results obtained by phenotyping-based biochemical assay systems and genotyping-based identification systems. Additionally, the methodology is very rapid and therefore well exemplifies a “rapid microbiological assay” (Gaudreau et al., 2018). 

Determination of the bacterial-cell chemical composition also plays a role in bacterial systematics (chemosystematics). Chemotaxonomic methods may be helpful in particular for classifying the bacterial taxa whose morphological and physiological characteristics vary widely and are insufficient for their satisfactory identification. Additionally, cell wall composition determines serological properties of bacteria. This principle underlies the immunochemical techniques for their identification.

Investigators sometimes also employ the lipid and fatty-acid composition of bacterial cells as chemotaxonomic markers. Active research on fatty acids has become possible with advancements in gas chromatography. Differences in the lipid profile are used for the identification of bacteria at species and genus levels. This method, however, has some limitations because fatty-acid content of cells may depend on cultivation conditions and culture age.

Analysis of nucleotide sequences of rRNAs has gained much popularity and importance for the identification of bacteria and for the creation of phylogenetic approaches to their classification

Founded at a federal publicly funded scientific institution, the Federal Research Center ICG SB RAS, a collection of biotechnologically important microorganisms contains more than 1500 strains, cultures of microorganisms, and DNA samples valuable for science and biotechnology and is intended for identification of new microorganisms with promise in terms of biotechnology and bioengineering and for studies on their genetics and metabolism. The collection contains representatives of all major superkingdoms (fungi, bacteria, archaea, and algae) and physiological groups (including anaerobes and extremophiles). Most strains in the collection have been isolated from previously unstudied unique extreme ecosystems: brine lakes, hot springs, soils, offshore areas, and bodies of freshwater

For accurate species level identification of microorganisms, currently, investigators are increasingly applying a combination of standard microbiological techniques of cultivation and visual observation with molecular biological and genetic methods, which help distinguish the species and strains of microorganisms at the level of DNA or RNA molecules (Kardymon, Kudryavtseva, 2016). For biotechnological purposes, it is important to have information not only about species affiliation of strains but also about their substrate specificity, completeness of metabolic pathways’ implementation, activity of metabolic reactions, and the possibility of their modulation. Consequently, an integrated approach to the identification of natural microorganisms will simplify the search for the strains that hold promise for bioengineering tasks. 

The purpose of this study was to identify microorganisms from the ICG SB RAS Collection via an integrated approach involving a combination of a wide variety of phenotypic and genotypic characteristics. 

## Methods

Phenotypic characterization. The shape and size of live and stained cells were determined using light and electron microscopes Axioskop 2 Plus, Axioskop А1, LIBRA120 (Carl Zeiss) at the Multi-Access Center for Microscopic Analysis of Biological Objects (SB RAS). The samples were prepared by standard methods (Netrusov et al., 2005). Gram staining was conducted by means of the Gram Stain Kit (Sintakon, Russia) according to the manufacturer’s instructions. 

The optimal temperature and pH for growth, NaCl tolerance, catalase and urease oxidase activities, anaerobic growth, amylolytic and caseinase activities, and other activities as well as the ability to utilize various substrates were determined according to Netrusov et al. (2005) and Logan & De Vos(2009). Most of the assays were carried out using Lachema and BioLog reagents and assay kits. 

Sequencing of 16S rRNA genes. Taxonomic affiliation (phylogenetic position) of the strains was determined according to the 16S rRNA gene sequence. To this end, bacterial DNA was isolated by the standard phenol method (Maniatis et al., 1984). Amplification of the 16S rRNA gene was conducted with universal bacterial primers 16S-8-f-B (5ʹ-AGR GTTTGATCCTGGCTCA-3ʹ) and 16S-1350-r-B (5ʹ-GAC GGGCGGTGTGTACAAG-3ʹ). The reaction mixture consisted of 1.5 mM MgCl2, 65 mM Tris-HCl (pH 8.8), 16 mM (NH_4_)2SO_4_, 0.05 % of Tween 20, 0.2 mM each dNTP, 0.3 mM each primer, and 1 U of recombinant Taq polymerase (SibEnzyme, Novosibirsk, Russia). DNA sequencing was performed by the Genomics Multi-Access Center, SB RAS.

Searches for similar sequences in nucleotide databases were conducted by means of the software of the Blast series (http://blast.ncbi.nlm.nih.gov/Blast.cgi). Sequence alignment was performed in the ClustalW software (http://www.ebi.ac.uk/Tools/msa/clustalw2)

Chemotaxonomic properties. To analyze the fatty-acid composition of cells, the strains were grown at optimal temperature until the exponential growth phase. The biomaterial obtained was processed according to (Jenkins, Tanner, 1977); after alkaline hydrolysis of lipids, acids were extracted with hexane and were methylated with a methanolic HCl solution according to Schäffer et al. (2002). The mixture of methyl esters of fatty acids was analyzed by gas chromatography on an Agilent Technologies 6890N chromatograph coupled with an Agilent Technologies 5973N quadrupole mass spectrometer and a quartz DB-1 column. The carrier gas was He at a constant flow rate of 1 ml/min. Injection temperature was 250 °С, and sample (1 µl) injection was performed via a microsyringe; electron impact ionization was set to 70 eV. Chromatographic-mass-spectrometric analysis of the studied solutions was conducted by means of total ion current in SCAN mode in the mass range 10 to 800 Da, with selective ion monitoring (SIM) of molecular ions of the compound being studied. Identification of methyl esters of fatty acids was performed through comparison with database NIST Mass Spectral Search Program for the NIST/EPA/NIH Mass Spectral Library Version 2.0a, build “Jul 2002.”

Mass-spectrometric analysis was conducted on an Ultraflex III MALDI TOF/TOF mass spectrometer (Bruker Daltonics). The spectra were recorded in linear positive mode at a laser frequency of 100 Hz in the mass range 2000–20000 Da. Accelerating electrode voltage was 25 kV, IS2 voltage 23.45 kV, and lens voltage 6 kV, without an extraction delay. For each sample, three spectra were acquired by summing up 500 laser impulses (5×100 impulses at different positions of the target cell). External calibration was performed with precise masses of known proteins: Escherichia coli RL36: 4365.3 Da, RS22: 5096.8 Da, RL34: 5381.4 Da, RL32: 6315.0 Da, RL29: 7274.5 Da, and RS19: 10300.1 Da. The series of spectra obtained for each strain were employed to generate the characteristic spectra in Biotyper 3.0 software, which constituted a list of mass peaks with averaged m/z values and relative peak intensities

To identify microorganisms from the ICG SB RAS Collection, the phenotypic and genotypic characteristics determined were analyzed in Statistica 6.0 software. Dendrograms were constructed by the method of unweighted pairwise arithmetic mean, and two-dimensional graphs by multidimensional scaling. Multivariate analysis was carried out in Past 3, version 3.25 (Hammer et al., 2001). In accordance with the requirements of the software, semiquantitative data on 95 substrates of the BioLog assay system were converted to numerical values: data on the absence/presence were coded as “0” or “1,” respectively, and undetermined values as “?”

## Results

In this study on microbial identification, 93 strains from the ICG SB RAS Collection were analyzed. Geographic locations of sampling for the isolation of microbial cultures were rather diverse: from vineyards of Crimea to geysers of Kamchatka and the Kuril Islands. Ecological types of habitats varied widely too: from bodies of freshwater to salty soils; temperature conditions were cold or thermal; environmental pH levels were neutral, acidic, or alkaline. The samples were collected both from unaffected natural areas of water and from anthropogenically polluted rock types

Strains were isolated on various media: e. g., LB, beef extract agar, beef-extract broth, or Pfennig’s medium with supplements. The cultivation was conducted at 32 to 55 °С. Each strain was subjected to phylogenetic, phenotypic, and mass-spectrometric characterization.

Morphology and biochemical properties

Most strains produced circular white, cream-colored, or yellow colonies. Colony edges were even or wavy, and the profile was flat or convex. Colony sizes varied from pinpoint size (less than 1 mm) to >5 mm. Cells of the strains were rodlike. Seventeen strains secreted a pigment into the medium. The cell wall was gram-positive. Streak growth varied among the strains: from nondiffuse to highly diffuse and from rosarylike to solid. Seventy-five strains featured sporogenesis.

The temperature ranges for growth tested were within 8–70 °С, and the pH ranges tested were within 2–10. The suitable temperature range for the growth of thermophilic microorganisms was 40–70 °С with an optimum at 60 °С. The suitable range for the growth of mesophilic microorganisms was 25 to 40, 50, and 55 °С, with an optimum mostly at 35 °С. Strong growth of the strains was noted at a NaCl concentration of 1 g/l. Some strains did not grow or grew poorly at 5 g/l NaCl in the medium. 

All the strains studied were tested for the ability to utilize various substrates by means of Lachema and BioLog assay systems. The strains were found to be aerobes and/or facultative anaerobes. In terms of nutrition, the strains turned out to be heterotrophs and chemoorganoheterotrophs. An overwhelming majority of the strains grew well on media with casein, starch, or Tween as a sole carbon source. Seventy-three strains showed a well-pronounced caseinase activity, characterized by the presence of clear zones around colonies after treatment with acetic acid (Netrusov et al., 2005). Furthermore, 81 strains had a good amylolytic activity, evidenced by clear zones. In a reaction with iodine, aside from the usual loss of color, in some cases, there was reddening of the medium around colonies, indicating the formation of dextrins.

It was determined that 40 strains possess a β-galactosidase activity. Virtually none of the strains utilized malonate, citrate, ornithine, or sulfur compounds (negative results of an H_2_S test). None of the strains except one utilized lysine, and 17 strains had a urease activity. None of the strains manifested a β-glucuronidase activity. The strains either utilized or did not utilize mannitol, trehalose, lactose, cellobiose, arginine, melibiose, sorbitol, salicin, raffinose, inositol, arabitol, adonitol, and dulcite. Twenty strains featured a β-xylosidase activity

Most of the strains studied did not utilize D-turanose, N-acetyl neuraminic acid, p-hydroxyphenylacetic acid, methyl pyruvate, D-fucose, L-fucose, L-rhamnose, D-aspartic acid, D-serine, glycyl-L-proline glucuronamide, mucic acid, chinic acid, D-saccharic acid, α-hydroxybutyric acid, β-hydroxyD,L-butyric acid, α-keto-butyric acid, or sodium butyrate.

It was found that an overwhelming majority of the strains belong to the genus Bacillus, and the others to the genera Anoxybacillus, Lysinibacillus, Geobacillus, Paenibacillus, Achromobacter, Agrobacterium, and Stenotrophomonas. 

Characteristic mass spectra of protein profiles were obtained for 83 strains from the collection. The results of phyloproteomic analysis were consistent with taxonomic affiliation of the strains, as determined by the sequencing of 16S rRNA genes. The results of the mass-spectrometric analysis complemented the existing set of characteristic mass spectra and may be useful for further identification of microorganisms in the cases where obtaining a quality DNA sample for sequencing is problematic. 

Analysis of the fatty-acid composition of the cell wall revealed the following fatty acids: saturated unbranched: myristic acid (С14:0); branched-chain acids: isomyristic (isoС14:0), isopentadecanoic (isoС15:0), anteiso-pentadecanoic (aC15:0), isopalmitic (isoС16:0), and anteiso-palmitic (aC16:0); and monounsaturated: palmitoleic acid (С16:1). The profile and ratio of fatty acids in the cell wall of bacteria are important traits for the identification of these microorganisms. 

## Discussion

**Fig. 1.**
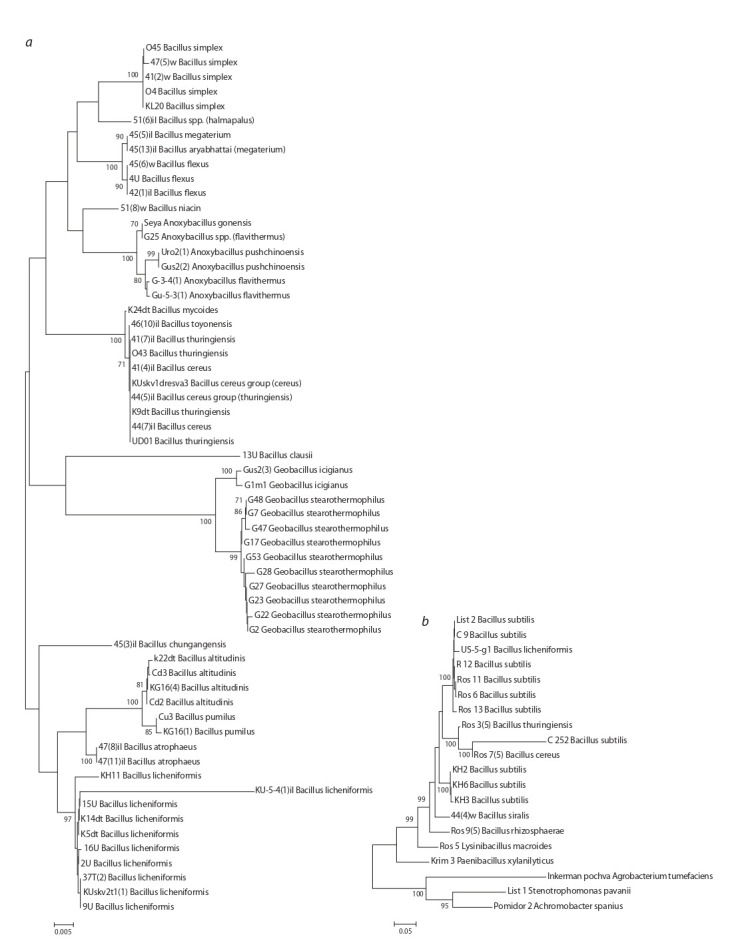
The phylogenetic tree constructed by the minimum evolution method applied to 16S rRNA sequences of the strains for which biochemical data were obtained by the Lachema assay (a) or BioLog assay (b). The numbers near clades denote bootstrap support.

**Fig. 2.**
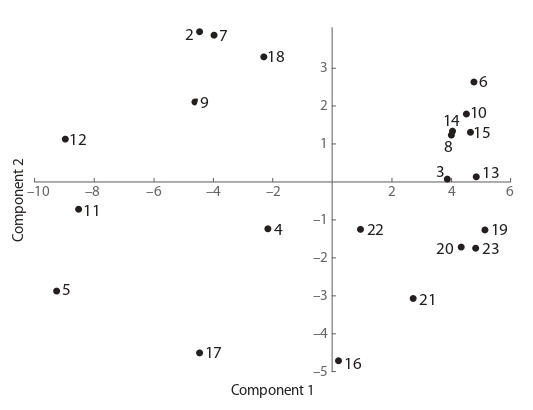
Results of the statistical analysis of the findings about the taxonomic profile of the microorganisms studied and their specific features of metabolism according to the first pair of principal components. The analysis covers 96 parameters.

Fig. 1 depicts a phylogenetic tree built from 16S rRNA sequences; it reflects the clustering of bacterial strains by species affiliation. Fig. 2 presents a statistical analysis of 21 strains for 96 formalized biochemical parameters determined by the BioLog Omnilog assays. This analysis did not reveal clear-cut clustering, especially judging by the strains of Bacillus subtilis (see Fig. 2). Ten strains from 21 samples analyzed belong to the species B. subtilis. Six of them (strains No. 10, 13, 14, 19, 20, and 21) are components of a relatively loose cluster that is formed by representatives of the B. subtilis group and B. cereus group. This cluster includes Lysinibacillus macrolides (strain No. 6). No other strains of B. subtilis (No. 2, 7, 11, and 17) clustered with their own species.

In prokaryotic systematics, for species identification, researchers utilize such parameters as rRNA sequence, cell membrane structure, and certain features of metabolism, e. g., methanogenesis or bacteriorhodopsin-dependent photosynthesis (DasSarma et al., 2019). Our results suggest that the features of metabolism analyzed are not species-determining but may play a major role when the usefulness of one or another strain for biotechnology is determined. This is because the possibility of degradation of various substrates is taken into account.

**Fig. 3.**
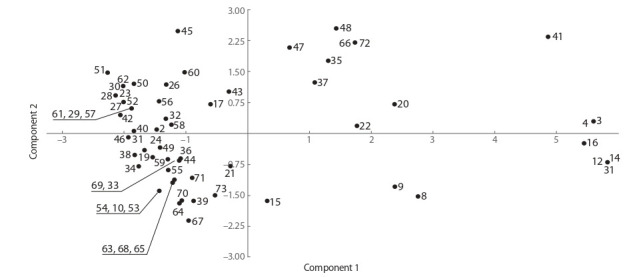
Results of the statistical analysis of the data on the taxonomic profile of the microorganisms studied and their specific features of metabolism according to the first pair of principal components. The analysis covers 29 parameters.

Fig. 3 shows the results of clustering of 61 strains by 29 parameters of metabolism, as determined by MICROLATEST® Lachema assays. We used such parameters as the ability of bacteria to utilize some sugars (e. g., mannitol, trehalose, lactose, cellobiose, sucrose, raffinose, and glucose), the presence of urease activity, and production of H_2_S. The analysis included strains of three Anoxybacillus species, 17 Bacillus species, and two Geobacillus species. Most of the strains formed a relatively tight cluster, regardless of the species of a microorganism, thus indicating a similarity between the substrates used for growth. The ability to grow on various sugars and organic acids (which is what most of the substrates tested were) is typical of the representatives of various taxonomic groups from the bacterial kingdom, irrespective of their origin. The second cluster (a smaller one) is formed by some strains of bacteria belonging to the following species: B. simplex, Anoxybacillus spp. ( flavithermus), G. stearothermophilus, B. mycoides, A. pushchinoensis, and B. licheniformis. These species were found to be represented by multiple strains, but the other strains of these species ended up in a large cluster according to metabolic characteristics. The unification of these strains into one cluster by metabolic characteristics points to a similarity between the substrates or possibly to the loss of the ability to utilize some of the substrates. In this case, the clustering does not reflect a common evolutionary origin, but rather shows substrate specificity that has developed as a consequence of convergent processes, probably during the adaptation to the substrates. According to the sampling sites, the species that ended up in the small cluster do not have a common origin either

**Fig. 4.**
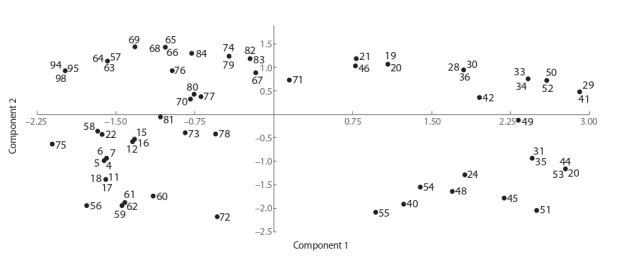
Results of the statistical analysis of the data on the taxonomic profile of the microorganisms studied and their specific features of metabolism according to the first pair of principal components. The analysis covers 29 parameters.

There was clustering by morphological characteristics too. For instance, the small cluster highlighted in Fig. 4 is formed by the strains of microorganisms that produced relatively large colonies. 

Evidently, further refinement and expansion of the spectrum of taxa and of their metabolic maps may lay the foundation for an “artificial” classification that may be helpful as a key for simplified and quicker identification and recognition of microorganisms. 

## Заключение

For 93 strains of microorganisms from the ICG SB RAS Collection, we determined key molecular-genetic and phenotypic characteristics. The strains were characterized by morphological, physiological, molecular-genetic, and mass-spectrometric parameters. Specific features of the strains’ growth on various media were determined, and cell morphology was assessed. Next, the strains were tested for the ability to utilize various substrates. It was found that the strains studied substantially differ in their biochemical characteristics. Additionally, we evaluated physiological characteristics of the strains from this collection: e. g., the relationship with oxygen, nutrition type, suitable ranges of temperature and pH, and NaCl tolerance.

Application of the integrated approach to microbial identification is necessary for solving the problems of targeted searches for biotechnologically promising strains. In this study, we combined microbes into separate groups/clusters on the basis of similarities of their phenotypic characteristics. This categorization, after further elaboration and expansion of the spectrum of taxa and of their metabolic maps, may form the basis for the creation of an “artificial” classification that may be helpful as a key for simplified and faster identification and recognition of microbes within both the ICG SB RAS Collection and other collections.

## Conflict of interest

The authors declare no conflict of interest.
